# Hepatitis C viraemic and seroprevalence and risk factors for positivity in Northwest Cambodia: a household cross-sectional serosurvey

**DOI:** 10.1186/s12879-021-05826-0

**Published:** 2021-02-26

**Authors:** Emily Lynch, Gregoire Falq, Chhorvy Sun, Pharm D Tek Bunchhoeung, Helena Huerga, Anne Loarec, Jean-Phillipe Dousset, Tonia Marquardt, Mickael Le Paih, David Maman

**Affiliations:** 1 Epicentre, 40 Rector St, New York, NY 10006 USA; 2 Epicentre, Paris, France; 3 Médecins Sans Frontières, Phnom Penh, Cambodia; 4 Cambodian Ministry of Health, Phnom Penh, Cambodia; 5 Médecins Sans Frontières, Tokyo, Japan

**Keywords:** Hepatitis C, HCV, Sero-prevalence, Cambodia, Viraemia, MSF, Serosurvey

## Abstract

**Background:**

Despite a dramatic reduction in HCV drug costs and simplified models of care, many countries lack important information on prevalence and risk factors to structure effective HCV services.

**Methods:**

A cross-sectional, multi-stage cluster survey of HCV seroprevalence in adults 18 years and above was conducted, with an oversampling of those 45 years and above. One hundred forty-seven clusters of 25 households were randomly selected in two sets (set 1=24 clusters ≥18; set 2=123 clusters, ≥45). A multi-variable analysis assessed risk factors for sero-positivity among participants ≥45. The study occurred in rural Moung Ruessei Health Operational District, Battambang Province, Western Cambodia.

**Results:**

A total of 5098 individuals and 3616 households participated in the survey. The overall seroprevalence was 2.6% (CI95% 2.3–3.0) for those ≥18 years, 5.1% (CI95% 4.6–5.7) for adults ≥ 45 years, and 0.6% (CI95% 0.3–0.9) for adults 18–44. Viraemic prevalence was 1.9% (CI95% 1.6–2.1), 3.6% (CI95% 3.2–4.0), and 0.5% (CI95% 0.2–0.8), respectively. Men had higher prevalence than women: ≥18 years male seroprevalence was 3.0 (CI95% 2.5–3.5) versus 2.3 (CI95% 1.9–2.7) for women. Knowledge of HCV was poor: 64.7% of all respondents and 57.0% of seropositive participants reported never having heard of HCV.

Risk factor characteristics for the population ≥45 years included: advancing age (*p*< 0.001), low education (higher than secondary school OR 0.7 [95% CI 0.6–0.8]), any dental or gum treatment (OR 1.6 [95% CI 1.3–1.8]), historical routine medical care (medical injection after 1990 OR 0.7 [95% CI 0.6–0.9]; surgery after 1990 OR 0.7 [95% CI0.5–0.9]), and historical blood donation or transfusion (blood donation after 1980 OR 0.4 [95% CI 0.2–0.8]); blood transfusion after 1990 OR 0.7 [95% CI 0.4–1.1]).

**Conclusions:**

This study provides the first large-scale general adult population prevalence data on HCV infection in Cambodia. The results confirm the link between high prevalence and age ≥45 years, lower socio-economic status and past routine medical interventions (particularly those received before 1990 and 1980). This survey suggests high HCV prevalence in certain populations in Cambodia and can be used to guide national and local HCV policy discussion.

## Background

Until very recently, the global Hepatitis C Virus (HCV) epidemic, responsible for an estimated 71 million chronic infections and 30% of the 1.34 million deaths due to viral hepatitis, was largely thought insurmountable [1]. Though newer Direct Acting Antivirals (DAA) are safer, more effective and easier for patients than previous HCV treatment, the extremely high treatment cost (up to $150,000 per patient) prohibited their widespread access and use, particularly outside of well-resourced health systems [1, 2].

However, recent developments are restructuring the public health approach to HCV. Two common DAAs, Sofosbuvir (Sof) and Daclatasvir (Dac), have reduced dramatically in cost (Sof was $84,000/treatment in 2013, and is only $85 in 2019) [3]. Adjusted models-of-care in low-resource settings are treating more patients, as effectively, for a fraction of previous prices (cost-per-cure in a 2018 Cambodian cohort dropped from $1172 to $370) [4]. Widespread access to treatment is becoming a more realistic goal, encouraging Ministries of Health (MoH) and donors in low-resource settings to expand HCV treatment as part of the global push towards the elimination of viral hepatitis by 2030, a goal adopted in 2016 by the World Health Assembly [1, 5].

The HCV burden in Cambodia has long been thought high. However, prevalence estimates in the general population are lacking. Several studies have attempted to understand the magnitude of HCV prevalence in Cambodia, but most have methodological limitations or focus on specific sub-populations rather than on the general population, with considerable variation in their estimates of prevalence rates [6–9]. Anecdotal clinical evidence has suggested increased HCV risk based on historic exposures to unsafe transfusions or routine medical practices, but this hypothesis has not been tested in the general population.

In 2016, the Cambodian Ministry of Health (MoH) and MSF established an HCV screening and treatment project in Phnom Penh, with expansion to Moung Ruessei hospital in Battambang Province in April 2018. By the end of the third quarter of 2018, MSF had screened 36,029 patients for HCV (24,756 Phnom Penh & 11,273 Moung Ruessei) and initiated Direct Acting Antiviral (DAA)-based treatment for 9731 patients (8757 Phnom Penh & 974 Moung Ruessei).

This study establishes a robust estimate of the HCV burden and seropositivity risk factors for the general population in three rural districts of northwestern Cambodia.

## Methods

### Study setting

The survey was conducted from April to August 2018 in the health operational district of Moung Ruessei, Battambang province, located in northwestern Cambodia near the border of Thailand. The three surveyed (administrative) districts included 175 villages, 20 communes, and 13 health center catchment areas. The area population consisted of an estimated 202790 individuals in 42072 households; village sizes ranged from 139 to 3979 inhabitants (29–822 households).^1^

### Study population, survey design and sample size

This was a cross-sectional population-based survey, using a multi-stage cluster design with probability proportional to size (PPS) and random sampling of villages (using ENA software version 2011) and random sampling of households (25 per cluster). All consenting adults 18 years and above (including visitors^2^) were eligible for inclusion in the survey. The sampling methodology enabled an oversampling of the population ≥45 years old to account for higher expected prevalence in this population.

Sample sizes were calculated using EpiInfo software, with an estimated 7% HCV prevalence among adults ≥45 years and 1.6% HCV prevalence among all adults ≥18 years, at 95% confidence, precision = 1.0% (≥45 years) and 0.8% (≥18 years), a non-response rate of 15%, and an average household size of 4.7. A total of 147 clusters were selected (123 clusters targeting the population ≥45 and 24 clusters for the population ≥18). The final sample size required 4784 individuals (1610 aged ≥18 and 3174 aged ≥45), 3628 households (577 including ≥18 and 3051 including ≥45).

### Data collection

Fifteen teams (1 surveyor, 1 nurse) administered face-to-face standardized, pre-piloted electronic questionnaires to households and individuals. Questionnaires included information on socio-demography, migration, knowledge of HCV prevention and treatment, and individual history of HCV exposure and risk factors. Data were entered and collected using electronic tablets and then exported to a secure Kobo platform.

### Statistical analysis

Statistical analyses were conducted using probabilities/sampling weights calculated for each stage of the sampling: village, household and individual. The sampling stratum considered the cluster, and analysis considered the finite population correction factor.

We conducted a multivariate analysis, accounting for the sampling design, to identify risk factors for HCV serological infection among the population ≥45 years.^3^

Risk factors for seropositivity identified a priori demographic variables (age, gender, occupation, education level, ID poor card status^4^), spatial variables (health centers catchment area, distance to Moung Ruessei referral hospital, distance to the health center from the catchment area), medical variables (history of blood transfusion and blood donation, history and location/provider type for medical injections, surgery and delivery, dental and gum treatment, type of contraception, miscarriage and abortion) and behavioral variables (tattoos, piercing, IV drug use, pedicures, manicures and frequenting of barbershops). The association between the seroprevalence and the explanatory covariates was quantified by fitting a linear multivariate regression model. The multivariate analysis retained variables from the univariate analysis with *p*-value less than 0.2. Estimates of the regression coefficients of the model and odds ratios with their standard error are presented. In the final model, ‘unknown’ levels of medical variables (history of blood transfusion and blood donation, surgery and dental and gum treatment) were few and are recoded as ‘none’. Statistical analyses were conducted using R version 3.4.1 (R Development Core Team, 2014). Accounting for the sampling design, the survey package (Analysis of Complex Survey Samples, Thomas Lumley) version 3.34 estimated parameters, including standard errors (Horvitz-Thompson-type standard errors are used everywhere in the survey package [10]. Confidence interval calculations usually used the scaled Chi squared distribution for the log likelihood from a binomial distribution [11]).

The list of villages and population data was provided by the Provincial Health Department 2016 and 2017 census data. Household lists (official household registers or notebooks) were provided by chiefs of villages and updated to include any new or temporary residents.

### Community engagement

Prior to the start of the survey, meetings were organized with local authorities at all levels to introduce the objectives of the survey and to discuss the timeline and request for support. Mobilisers (identified by the chief of each village) visited selected households prior to the data survey to request the household’s presence.

### Laboratory procedures

Sero-infection was assessed for all participants using the SD Bioline® HCV rapid diagnostic test [12], performed according to the manufacturer’s instructions, using capillary blood collected by fingerpick by trained nurses.

Seropositive participants were invited to the nearest health center to assess their HCV viral load; HIV and HBV diagnostics were also offered to ensure smooth linkage to care but results were not tracked. Specimens were stored and transported to the MSF laboratory in Moung Ruessei hospital in cold chain (2–8 °C). Samples were centrifuged the same day and stored in a refrigerator (2–8 °C) before their analysis within 24 h. Viral load was assessed using the Xpert© HCV viral load assay with GeneXpert© Instrument Systems (Cepheid, Sweden).

### Linkage to treatment and care

Patients with detectable viral load were invited to the MSF/MoH HCV program at Moung Ruessei hospital to receive their results and initiate treatment, if desired (the survey reimbursed transport costs). Besides initial and final visits at the hospital, care was provided at the closest health facility to the patient’s home.

### Ethical considerations

This study received ethical approval from the MSF Ethical Review Board (ID: 1816), as well as the Cambodian National Ethics Committee for Health Research (NECHR; 23 February 2018 NECHR minutes).

## Results

### Participation

A total of 5098 (of 5215) individuals and 3616 (of 3668) households participated in the survey, with an individual and household response rate of 97.8 and 98.6%, respectively. The percentage of households absent or refusing was equal, at 0.7%, as was the percentage of individual absenteeism or refusal, at 1.1%.

### Study population

This section extrapolates the demographics of the surveyed population to the population as a whole to confirm the representativeness of the survey sample. The surveyed individuals and households represented a population of 112398 inhabitants living in Moung Ruessei district consisting of 49903 males and 62494 females, with an overall sex ratio of 0.80 (Table 1). This age and gender distribution in the study was similar to the population as a whole.^5^

**Table 1:** Weighted study population count and proportion, per sex and per age category

Age group	Male		Female		Total		Sex ratio
	N	%	N	%	N	%	
[18–24]	8591	17.2	8302	13.3	16,892	15.0	1,03
[25–34]	11,767	23.6	14,149	22.6	25,916	23.1	0,83
[35–44]	8663	17.4	10,540	16.9	19,203	17.1	0,82
[45–54]	7816	15.7	10,777	17.2	18,593	16.5	0,73
[55–64]	6828	13.7	9941	15.9	16,769	14.9	0,69
[65–74]	4325	8.7	5978	9.6	10,304	9.2	0,72
[75-Inf]	1914	3.8	2807	4.5	4721	4.2	0,68
Total	49,903	100.0	62,494	100.0	112,398	100.0	0,80

The percentage of the study population never attending school was ~ 50%, irrespective of gender (Table 2). Women achieved more education than men (24.6% of women have attended higher than secondary school, versus 10.3% of men).

**Table 2:** Description of the weighted study population

	Male		Female		18–44		45+		Total	
	N	%	N	%	N	%	N	%	N	%
**Education level**										
Never Attended	25.187	50.5	31.804	50.9	29.814	48.1	27.176	53.9	56.991	50.7
Primary	12.151	24.4	8702	13.9	13.933	22.5	6921	13.7	20.853	18.6
Secondary	7414	14.9	6610	10.6	11.911	19.2	2112	4.2	14.024	12.5
Higher	5152	10.3	15.378	24.6	6353	10.2	14.177	28.1	20.53	18.3
**Occupation**										
Farmer	31.634	63.4	28.683	45.9	31.98	51.6	28.337	56.2	60.317	53.7
Laborer	2768	5.5	2281	3.6	3898	6.3	1150	2.3	5049	4.5
Small Business	2560	5.1	5639	9	4837	7.8	3363	6.7	8200	7.3
Factory Worker	84	0.2	361	0.6	433	0.7	12	0	445	0.4
Student	1011	2	1733	2.8	2743	4.4	0	0	2743	2.4
Housework	964	1.9	10.664	17.1	5920	9.6	5708	11.3	11.627	10.3
Construction	1346	2.7	84	0.1	1227	2	202	0.4	1430	1.3
Cleaning/Maid	0	0	84	0.1	72	0.1	12	0	84	0.1
Pensioner	727	1.5	1085	1.7	72	0.1	1740	3.4	1812	1.6
None	2536	5.1	5586	8.9	1805	2.9	6318	12.5	8123	7.2
Other	6273	12.6	6295	10.1	9024	14.6	3545	7	12.568	11.2
**ID poor card program**										
ID poor card 1	5375	10.8	8292	13.3	5920	9.6	7748	15.4	13.667	12.2
ID poor card 2	7556	15.1	10.923	17.5	9313	15	9167	18.2	18.479	16.4
Poor letter	204	0.4	132	0.2	217	0.3	119	0.2	335	0.3
Not part of a poor program	35.665	71.5	42.263	67.6	45.119	72.8	32.81	65.1	77.929	69.3
Dont Know	1103	2.2	885	1.4	1444	2.3	543	1.1	1987	1.8

For both men (63.4%) and women (45.9%), farming was the predominant (53.7%) occupation, followed by small business (7.3%) and labor (4.5%) (Table 2).

Roughly a third (28.9%) of the population was part of a social welfare program (Table 2).

More than a quarter of the population was away from home sometime within the previous year (Table 3). The percentage of the population away for 1 month or longer was substantial: 9.0% were away 1 to 6 months and 7.0% were away more than 6 months.’(Table 3).

**Table 3:** Migration pattern in the study population per sex and per age category

	Male		Female		18–44		45+		Total	
	N	%	N	%	N	%	N	%	N	%
**Time away from home**										
Never absent	34,443	69.0	46,872	75.0	40,715	65.7	40,599	80.6	81,314	72.3
Less than 1 month	6024	12.1	7087	11.3	6208	10.0	6903	13.7	13,111	11.7
1 to 6 months	5509	11.0	4608	7.4	8157	13.2	1959	3.9	10,117	9.0
More than 6 months	3928	7.9	3927	6.3	6930	11.2	925	1.8	7855	7.0
**Reason to be away from home**										
Find a job	8765	92.9	7481	87.7	13,933	92.3	2314	80.2	16,246	90.4
Study	168	1.8	289	3.4	433	2.9	24	0.8	457	2.5
Other	503	5.3	766	9.0	722	4.8	547	18.9	1269	7.1

## Prevalence

### Main findings

**Fig. 1:**
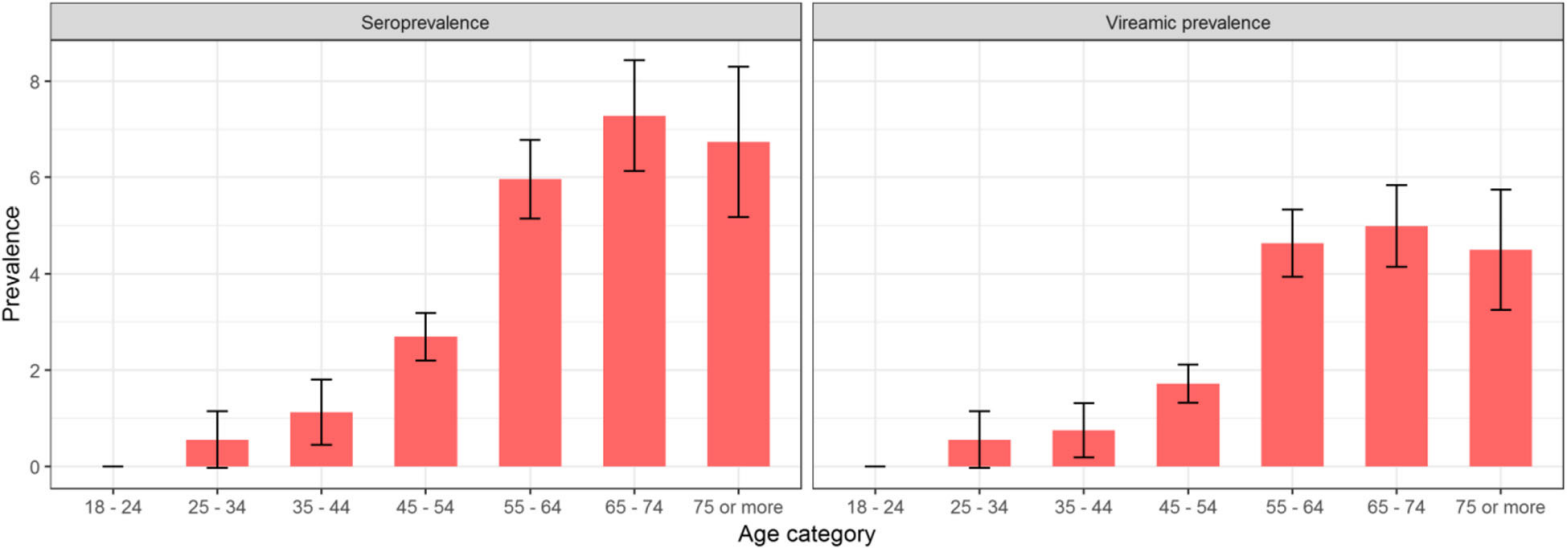
HCV seroprevalence and viraemic prevalence by age categories with 95% confidence intervals

The overall seroprevalence for the entire adult population ≥18 years was 2.6% (CI95% [2.3–3.0]) (Table 4), with 5.1% (CI95% [4.6–5.7]) in adults ≥45 years, and 0.6% (CI95% [0.3–0.9]) in adults 18–44. For ages 55–64, 65–74 and ≥75, the prevalence was, respectively, 6.0 (CI95% [5.2–6.8]), 7.3 (CI95% [6.1–8.4]), and 6.7 (CI95% [5.2–8.3]) (Fig. 1). Men had a higher prevalence than women: 3.0 (CI95% [2.5–3.5]) vs. 2.3 (CI95% [1.9–2.7]) (Table 4).

**Table 4:** Seroprevalence and viraemic prevalence per sex and per age category

	Serology		Viraemia	
	N	Prevalence CI95%	N	Prevalence CI95%
Overall	221/5098	2.61 [2.25–2.96]	157/5098	1.88 [1.62–2.14]
[18–44]	5/859	0.58 [0.27–0.89]	4/859	0.47 [0.17–0.76]
[45+]	216/4239	5.10 [4.55–5.65]	153/4239	3.62 [3.22–4.01]
Male	112/2159	3.03 [2.54–3.52]	84/2159	2.37 [1.94–2.79]
Female	109/2939	2.27 [1.87–2.66]	73/2939	1.49 [1.22–1.76]

Viraemic prevalence was also notably high in older adults. The prevalence for adults ≥18 years was 1.9 (CI95% [1.6–2.1]) (Table 4) and for adults ≥45 years it was 3.6 (CI95% [3.2–4.0]), while it was 0.5 (CI95% [0.2–0.8]) in adults 18–44. Like the serological results, men had higher viraemic prevalence than women, at 2.4 (CI95% [1.9–2.8]) compared to 1.5 (CI95% [1.2–1.8]), however, with overlapping confidence intervals.

### Geographical pattern

**Fig. 2:**
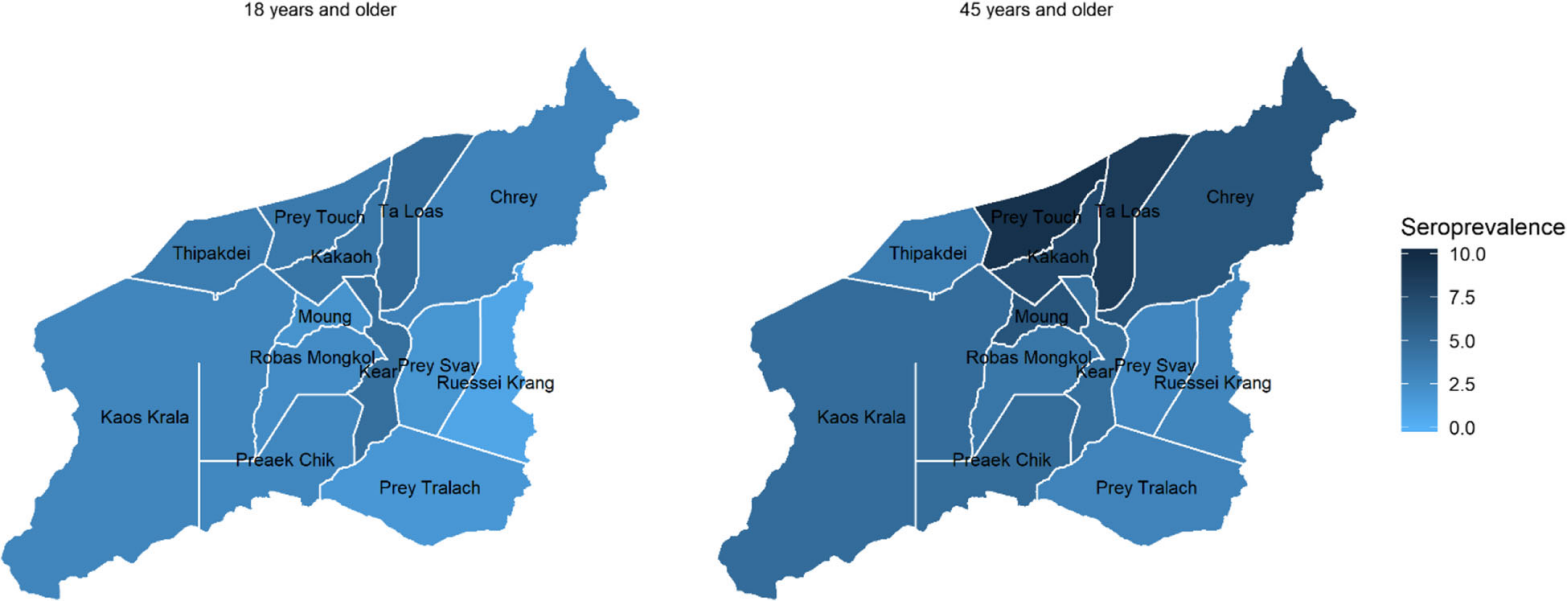
HCV serological prevalence by health center catchment area

Among adults ≥45 years old, geographic variation in seroprevalence was noted at the level of the health center catchment area, ranging from the lowest prevalence, in Prey Svay, of 3.0 (CI95% [1.3–4.6]) to the highest in Prey Toch, at 9.4 (CI95% [6.7–12.1]) (Table 5). Nevertheless, this analysis was unable to quantify any clear geographic patterns in seroprevalence. Exploratory analyses showed no identifiable trends in prevalence according to the distance from the catchment area to Moung Ruessei referral hospital, or to the health center (Fig. 2).

**Table 5:** HCV seroprevalence per health center catchment area by age categories

	≥ 18 years		≥ 45 years	
	N	Prevalence	N	Prevalence
Chrey	23/443	3.17 [2.13–4.20]	21/324	6.49 [5.21–7.77]
Kea	16/351	4.59 [2.46–6.71]	16/351	4.59 [2.46–6.71]
Keas Kralor	17/391	2.99 [1.75–4.24]	16/324	4.82 [3.30–6.33]
Kor Kos	23/360	4.13 [3.28–4.97]	22/295	7.57 [4.58–10.55]
Mong	27/586	2.02 [1.05–3.00]	26/392	6.56 [4.15–8.97]
Prek Chik	14/321	2.86 [1.51–4.20]	14/288	4.86 [3.54–6.19]
Prek Tralach	16/578	1.75 [1.09–2.42]	16/513	3.11 [2.35–3.87]
Prey Svay	11/414	1.87 [1.20–2.55]	11/378	2.95 [1.28–4.63]
Prey Toch	17/225	3.87 [0.83–6.90]	17/182	9.41 [6.72–12.09]
Robos Mongkol	15/405	2.57 [1.25–3.89]	15/370	4.05 [2.81–5.28]
Russei Kraing	10/500	0.74 [0.51–0.97]	10/335	2.99 [2.14–3.83]
Talars	25/329	4.82 [3.46–6.18]	25/292	8.56 [6.98–10.15]
Thitpdey	7/195	3.59 [1.58–5.60]	7/195	3.59 [1.58–5.60]

### Knowledge of HCV

Most participants, 3302/5103 (64.7%), reported never having heard of HCV.^6^ This percentage was lower amongst seropositive participants (57.0%). Among those who reporting having heard of HCV, knowledge of HCV transmission was similarly reported between seropositive and seronegative participants (55.8% versus 53.2%). The percentage of all survey participants who had both heard of HCV and accurately reported the transmission pathways ranged from 12.6 to 17.5%. Among the 1801 participants who had heard of HCV, 1139 participants (63.2%) responded that they know how HCV can be prevented. Although most participants correctly reported HCV prevention methods (“not sharing needles or syringes with other people,” “using a condom,” and “using sterile or unused medical devices”: 93.1, 80.0, 88.2%, respectively), a majority (85.4, 82.2%) also reported that “getting a vaccine,” and “washing your hands thoroughly,” were other ways to prevent transmission.

### Seropositivity risk factor analysis

Table 6 presents socio-demographic characteristics and risk factors associated with seroprevalence in the multivariate linear regression model (accounting for the sampling design) for adults ≥45 years.

**Table 6:** Socio-demographic characteristics and risk factors, adults ≥45 years old (*p*-value per level, global *p*-value and odds ratio)

		*p*-value	Globalp-value	Adusted OR (CI95)	unadusted OR (CI95)
	age	–	< 0.001	1.04 [1.03–1.04]	1.03 [1.03–1.04]
Level of education Reference: Never Attended	Primary	0.070	< 0.001	1.20 [0.99–1.45]	1.31 [1.09–1.58]
	Secondary	0.757		1.05 [0.76–1.45]	1.21 [0.87–1.67]
	Higher	< 0.01		0.68 [0.55–0.84]	0.75 [0.61–0.91]
ID poor card Reference: Not part of a poor program	ID poor card 1	< 0.05	< 0.01	0.76 [0.59–0.98]	0.69 [0.53–0.89]
	ID poor card 2	0.225		1.13 [0.93–1.38]	1.03 [0.86–1.25]
	Poor letter	0.063		2.38 [0.97–5.87]	2.00 [0.76–5.26]
	Don’t Know	0.056		1.97 [0.99–3.91]	1.72 [0.89–3.32]
Health center Reference = Chhrey	Kea	< 0.05	< 0.001	0.59 [0.35–0.99]	0.69 [0.40–1.19]
	Keas Kralor	0.137		0.72 [0.47–1.11]	0.75 [0.50–1.11]
	Kor Kos	0.342		1.26 [0.78–2.03]	1.16 [0.72–1.88]
	Mong	0.582		0.88 [0.56–1.38]	1.02 [0.65–1.62]
	Prek Chik	0.237		0.80 [0.56–1.15]	0.74 [0.51–1.06]
	Prek Tralach	< 0.001		0.47 [0.34–0.65]	0.46 [0.33–0.65]
	Prey Svay	< 0.01		0.43 [0.23–0.79]	0.43 [0.23–0.82]
	Prey Toch	0.062		1.46 [0.99–2.17]	1.49 [1.01–2.18]
	Robos Mongkol	< 0.01		0.57 [0.38–0.85]	0.61 [0.41–0.90]
	Russei Kraing	< 0.001		0.46 [0.32–0.65]	0.44 [0.31–0.64]
	Talars	0.084		1.28 [0.97–1.70]	1.35 [1.00–1.82]
	Thitpdey	< 0.05		0.49 [0.25–0.95]	0.54 [0.29–1.01]
Injection – threshold 1990 Reference = injection before 1990	No injection	0.157	< 0.05	0.78 [0.56–1.10]	0.62 [0.45–0.86]
	Injection 1990 or after	< 0.01		0.74 [0.62–0.89]	0.58 [0.48–0.69]
	Don’t Know if injection	0.553		0.70 [0.22–2.26]	0.79 [0.24–2.58]
Surgery – threshold 1990 Reference = Surgery before 1990	No surgery	< 0.001	< 0.001	0.51 [0.38–0.68]	0.39 [0.30–0.51]
	surgery 1990 or after	< 0.01		0.66 [0.49–0.88]	0.51 [0.39–0.68]
Blood donation – threshold 1980 Reference = blood donation before 1980	No blood donation	< 0.01	< 0.01	0.35 [0.19–0.66]	0.25 [0.14–0.44]
	blood donation before 1980	< 0.05		0.43 [0.22–0.84]	0.30 [0.16–0.55]
Blood transfusion – threshold 1990 Reference = blood transfusion before 1990	No blood transfusion	< 0.01	< 0.01	0.53 [0.35–0.80]	0.31 [0.20–0.46]
	Blood transfusion 1990 or after	0.104		0.66 [0.41–1.08]	0.44 [0.27–0.71]
Dental and Gum treatment Reference = No	Yes	< 0.001	< 0.001	1.56 [1.33–1.84]	1.70 [1.45–1.99]

Seroprevalence increased with age (*p*< 0.001) and was associated with socio-economic status, being lower among people who achieved an education level higher than secondary school than those who never attended school (OR 0.7 [95% CI 0.6–0.8]); there was also some evidence that seroprevalence was lower among people with an ID poor card 1 compared to those not part of a welfare program (OR 0.8 [95% CI 0.6–1.0], *p*< 0.05). Seroprevalence was higher among people who had their first medical injection before 1990 (injection after 1990 OR 0.7 [95% CI 0.6–0.9]),their first surgery before 1990 (surgery after 1990 OR 0.7 [95% CI 0.5–0.9]); people who donated blood for the first time before 1980 (blood donation after 1980 OR 0.4 [95% CI 0.2–0.8]),a blood transfusion for the first time before 1990 (blood transfusion after 1990 OR 0.7 [95% CI 0.4–1.1]); and those who had dental and gum treatment (OR 1.6 [95% CI 1.3–1.8]). The degrees of freedom of the model was high (30) given the number of events (216). Nevertheless, the *p*-value of the Hosmer Lemeshow test that assesses the goodness-of-fit of the model was less than 0.05.

## Discussion

This survey is the first of its kind in size and rigor in Cambodia, with findings that: HCV prevalence is higher in people ≥45 years (with prevalence increasing with older age) and among those with less education, that there is sometimes wide geographic variability in HCV estimates, and that HCV disease is poorly understood even among the seropositive.

The prevalence rates found were similar to estimates from some previous studies, but substantially different from others. The 2.6% prevalence seen in this population was far lower than the 14.7% seen in blood donors in 2009, and was half the rates (5.8 and 5.2%) from observational studies of people living with HIV (PLHIV) [7–9]. The only other survey of a general population cohort, from Siem Reap in 2012 (across only three northern villages; *n*=483), found double (5.2%) the seroprevalence of this cohort, though our survey confirmed several findings of that survey as well (higher prevalence in older cohorts, geographic variability in seroprevalence, similar viraemic prevalence) [13]. Notably, our results diverge from that smaller survey in the gender differences in seropositivity risk that we found, and the fact that the Siem Reap survey associated blood transfusions and surgical history with a higher risk of viremia, while ours associated these factors with seroprevalence.

Surveys in nearby Thailand have similarly found a wide range of HCV prevalence among different regions and groups. A recent large-scale survey of the general population in Phetchabun of adults aged 35–64 found 6.9% anti-HCV positive [14], though an earlier study found a 15.5% anti-HCV positivity rate in Phetchabun, compared to 3.6% in neighboring Khon Kaen Province [15]. More similarly to our results among older adults (but dissimilarly as it was among a sub-group), a 5.5% HCV seroprevalence was identified among HIV cohorts in Cambodia in a systematic review and meta-analysis [16].

Predictably, HCV seroprevalence was especially linked (among those ≥45 years) to routine medical care (dental), and procedures occurring prior to 1990 and 1980: injections, surgery, blood donation and transfusion. Though these findings may speak to historic trends, the fact that the study cannot determine when transmission occurred in this population means it is critical to generally reinforce infection prevention and control (IPC) measures in healthcare facilities and among staff at all levels.

Furthermore, programs for HCV screening and treatment should particularly consider older populations. Systematic testing of those aged ≥45 years, and treatment for the viraemic positives would contribute to the goal of HCV elimination in Cambodia and could substantially reduce the existing reservoir of HCV in the general population. Increasing overall awareness of HCV in the general population through information, education and communication (IEC) will also be a critical component of decreasing transmission and prevalence.

There have been many obstacles on the road to HCV elimination. For years, effective drugs were lacking, and then cost-prohibitive. Now, simplified care models make HCV interventions more efficient and cost-effective. Establishing baseline prevalence rates and identifying groups at highest risk for seropositivity using statistically rigorous methods like the ones described here provides the necessary evidence for HCV elimination programs.

The results of this study are sufficiently representative, demographically and geographically, to provide policy-relevant guidance on HCV screening strategies, treatment and elimination in Cambodia, and to contribute to global conversations on HCV epidemiology, treatment and disease reduction or elimination [13].

## Limitations

There are several limitations to this study. There were not enough seropositive participants under 45 years to conduct a meaningful analysis of risk factors for infection in this age group. Also, children were not included. The timing of initial HCV infection cannot be identified and therefore it is not possible to describe historical HCV epidemiology from this survey.

Although the study ensured that all participants were provided with transport for follow-up testing and to initiate care and treatment, the study design did not maintain data regarding uptake of care or initiation of treatment. This decision was made after deliberation and taken in light of ethical concerns that participants could feel unfairly pressured to participate in the survey if they were to be followed through the course of their decision regarding treatment initiation, since MSF both conducted the survey and, in collaboration with the MOH, provided treatment.

Moreover, the survey is not representative of all Battambang Province or of other regions in Cambodia, and the surveyed geographic area may have had unique characteristics (such as a low distance [< 20 km] from most health centers to the hospital or largest town) and should not be considered representative of other regions. Care should be taken when interpreting results, though these findings point to important potential infection trends and are the most robust rural HCV estimates in the country to date.

## Conclusion

This study provides the first large-scale general adult population prevalence data on HCV infection in Cambodia. The primary conclusions fill gaps in the understanding of HCV epidemiology in Cambodia with more precision and power than currently existing data. The results show high prevalence in adults age ≥45 years and confirm the link between high prevalence and increasing age, lower socio-economic status and past routine medical interventions (particularly those before 1990 and 1980). This survey serves as an alert to the potentially high prevalence of HCV infection in Cambodia and can be used to guide national and local HCV policy discussion.

## Data Availability

The datasets used and/or analysed during the current study are available from the corresponding author on reasonable request.

## Abbreviations

CI: Confidence interval
DAA: Directly acting antivirals (drug)
ENA: Emergency nutrition assessment software
ERB: Ethical review board
HBV: Hepatitis B virus
HCV: Hepatitis C virus
HIV: Human immunodeficiency virus
IEC: Information education and communication
IPC: Infection prevention control
MOH: Ministry of health
MSF: Médecins Sans Frontières
NECHR: National Ethics Committee for Health Research
OR: Odds ratio
PLHIV: People living with HIV/AIDS

## Footnotes

1 Census 2016, Cambodian Ministry of Planning

2 Defined as any person who slept in the household the previous night

3 We focused on this older cohort for the risk factor analysis both because 1) in the ≥ 18 age group there were very low rates of positivity and, among those positive, insufficient numbers of affirmative responses in the respective risk factor categories to enable meaningful analysis and 2) the ≥45 years population was of primary programmatic and advocacy interest due to the assumption (justified by the results of this survey) of the elevated comparative prevalence in this population as compared to the younger cohort.

4 The ID poor card is a system in Cambodia to identify impoverished households eligible to receive public assistance. For the purposes of this survey, there were three possible types of ID poor card status: 1) ID poor card 1, 2) ID poor card 2 and 3) Poor letter (whereby the village chief or another local leader provided a letter confirming the impoverished status of the family). The difference between ID poor card 1 and 2 is a matter of severity of poverty levels; the poor letter is written in the absence of having an official designation as ID poor card 1 or 2, for example in sudden or unexpected circumstances

5 Department of Economic and Social Affairs, 2017

6 Knowledge of HCV was assessed by the following questions: 1) Have you ever heard of Hepatitis C Virus or HCV? 2) Do you know how the hepatitis C virus is transmitted? 3) Can Hepatitis C virus be transmitted through: droplets (coughing, sneezing, respiratory secretions)? Food? Blood? Sexual contact? Handshake with an infected person? Sharing household objects like razors or toothbrushes? Sharing needles or syringes? touching items in public places (doorknobs, handles in transport, unhygenic toilets)? 4) Are there any other ways Hepatitis C virus can be transmitted that I haven’t mentioned? Please specify. 5) Next I am going to ask you a few questions about the disease that is caused by hepatitis C virus. Please answer based on what you already know. Is it possible to have the hepatitis C virus but not have any symptoms? 6) Are there medications available to treat Hepatitis C virus infections? 7) Do you know how the hepatitis C can be prevented? 8) Can Hepatitis C virus infection be prevented by: getting a vaccine? using a condom? not sharing needles or syringes with other people? washing your hands thoroughly? using sterile or unused medical devices? 9) Are there other ways to prevent Hepatitis C virus infection that I haven’t mentioned? Please specify.
